# Effectiveness of Cold Smoking on Inactivating Murine Norovirus in Salami-Like Pork Sausages (Mettwurst), and Hepatitis E Virus and Murine Norovirus in Solution

**DOI:** 10.1007/s12560-024-09631-1

**Published:** 2025-01-10

**Authors:** Emil Loikkanen, Antti Mikkelä, Suvi Joutsen, Pirkko Tuominen, Leena Maunula

**Affiliations:** 1 Department of Food Hygiene and Environmental Health, Faculty of Veterinary Medicine, University of Helsinki, Helsinki, Finland; 2 Risk Assessment Unit, Laboratory and Research Department, Finnish Food Authority, Helsinki, Finland

**Keywords:** Murine norovirus, Hepatitis E virus, Cold smoking, Long-term virus inactivation, Salami-like sausage, Weibull model

## Abstract

Hepatitis E virus (HEV) is a zoonotic virus that infects humans when virus-containing pork products are consumed. This study aimed to explore MNV (murine norovirus) and HEV inactivation during cold smoking and ripening/fermentation treatments used for salami-like sausages (mettwurst). MNV inactivation was monitored in culture medium solution and in sausage while being subjected to a salami-like sausage manufacturing process. The inactivation of MNV in the solution was also monitored at room temperature (RT) for four weeks. HEV inactivation was monitored in solution during the cold smoking process and at RT. A TCID_50_ assay was used to calculate the infectious MNV and HEV titres. MNV survival was modelled using Bayesian inference. MNV load in solution decreased by as much as 4.7 (SD 0.9) log_10_ TCID_50_/ml when it was subjected to the cold smoking process. Modelling revealed that 99.999% MNV might be inactivated during the treatment when observed at a level of 95% CI (Bayesian Confidence Interval). On the contrary, MNV load decreased by only 1.8 (0.2) log_10_ when stored at RT. The low-titre HEV in solution was inactivated (> 1.1 (0.2) log_10_) when treated, and at RT. However, MNV was resistant in the sausage matrix during the cold smoking process (log_10_ reduction of 1.9 (0.5) TCID_50_/ml). Based on modelling, a substantial amount of virus would remain in the product, even when the uncertainty was considered. Hence, viruses, here exemplified by MNV, may not be inactivated from salami-like pork sausages during manufacturing, which poses a risk for consumers in real-life situations.

**Supplementary Information:**

The online version contains supplementary material available at 10.1007/s12560-024-09631-1.

## Introduction

There are approximately 20 million incidents of hepatitis E infections in the world annually, of which 3.3 million have symptoms and 44,000 lead to death (WHO, 2024). While all hepatitis viruses can be transmitted through blood, hepatitis A and hepatitis E virus (HEV) are often transmitted via food consumption. An alarming increase in HEV cases in Europe was observed during 2005–2015 based on reports from 22 EU/EEA countries (EFSA, 2017). Foodborne transmission appeared to be the major route.

Hepatitis E virus is a 32–34 nm icosahedral virus with an RNA genome of 7.2 kb, and belongs to the *Hepeviridae* family genus *Paslahepevirus* (Ahmed & Nasheri, 2023; Purdy et al., 2022). Specifically, *Orthohepevirus A* group includes genotypes 1–8 (HEV-1–HEV-8), of which HEV-1 through 4, as well as HEV-7 infect humans (Smith et al., 2013). The most common zoonotic HEV strains belong to HEV-3, which has main reservoirs in pigs and wild boars worldwide, whereas the occurrence of HEV-4 is more regional. HEV-3 can be further divided into recognized subtypes a–m (Smith et al., 2020). In addition, rare human cases caused by rat HEV strains, *Rocahepevirus ratti*, (earlier *Orthohepevirus C)* have emerged in Asia (Sridhar et al., 2018), and recently rat HEV has also been found in pigs in Spain (Rios-Muñoz et al., 2024).

Among blood donors in the Finnish population, the HEV IgG prevalence has been reported to be 7.4% (Mättö et al., 2023), while a prevalence of 10% total HEV antibodies was detected among veterinarians (Kantala et al., 2017). Among animals in Finland, 18% of wild boars living in nature had HEV antibodies (Fredriksson-Ahomaa et al., 2020). HEV seroprevalence was 9.1% in moose and 1.4% in white tailed deer (Loikkanen et al., 2020). In the study by Kantala et al. (2013) on Finnish pigs, HEV seroprevalence was detected to be over 80% at slaughter.

HEV-3 infection is often self-limiting and asymptomatic in humans (Guillois et al., 2016). However, it may lead to fulminant hepatitis or to neurological symptoms, as reviewed by Lhomme et al. (2020). Chronic infections caused by HEV-3 have been reported in immunocompromised patients (Narayanan et al., 2019; Pisano et al., 2021). HEV infection in pigs is generally considered asymptomatic, as also recently reviewed by Meester et al. (2021).

In the early 2000s, hepatitis E infections were rare in Finland. They were mostly travel-related and caused by HEV-1 (Kantala et al., 2009). However, in recent years, domestic HEV-3 cases have become more frequent; a case report presented by Kettunen et al. (2013) provides an example. Furthermore, during January–May 2024, over 200 persons with acute hepatitis E infections were reported from different parts of Finland. Based on trawling interviews, mettwurst was suspected as the source of infection (Personal communication: R. Rimhanen-Finne, 6.6.2024). Colson et al. (2010) had described a foodborne HEV outbreak caused by consumption of wild boar liver containing figatelli sausage in France. Furthermore, the study by Said et al. (2014) showed an association between domestic HEV infection and consumption of pork products in the UK.

Pig liver products in Europe have often been found to contain HEV RNA (Bouwknegt et al., 2007; Di Bartolo et al., 2012). For example, Di Bartolo et al. (2015) found the presence of HEV RNA in both raw (22.2%) and dry (4.3%) pig liver sausages. Infectious HEV has also been demonstrated in pig liver (e.g. Berto et al., 2013; Hakze-van der Honing et al., 2023). According to the review of Di Cola et al. (2021), during 2003–2018, there were reports of HEV-3, HEV-4, or HEV-7 outbreaks caused by contaminated pork meat, entrails, or spit-roasted piglet (5 outbreaks), wild boar meat, or raw bile juice from a wild boar (4), figatelli (4), deer meat (1), tap water (1), camel milk (1), and shellfish (1). The presence of HEV RNA in sausages has been demonstrated in several studies (Boxman et al., 2019, 2020; Moor et al., 2018; Szabo et al., 2015) and reviewed by EFSA (2017). In the study by Boxman et al. (2020), a high prevalence of HEV was reported in salami-like sausages, such as in Dutch mettwurst ‘metworst’ (26.1%) and salami (18.5%).

There are a variety of different types of non-thermally prepared sausages, especially in Europe. It is common to have regional habits on how they are prepared, in terms of ingredients and treatments. Traditionally, the used treatments (e.g. salt, smoking treatment, acidification by adding lactic acid bacteria, curing, and minimizing water content) are aimed at preventing growth of bacteria or fungi, and prolonging the storage time. Studies that simulate the preparation of fermented sausages have focussed on the effect of salt and pH on virus inactivation (Wolff et al., 2020a, 2020b). However, virus stability in salami-like sausages and the factors affecting it are still largely unknown.

HEV can survive for long periods at optimal temperatures, e.g. for over 56 days at 4 °C and over 20 days at room temperature (RT) when stored in solution (cell culture medium; Johne et al., 2016). Among the preventive measures of foodborne transmission, thermal stability of HEV has been the most intensively investigated, as reviewed by Johne et al. (2024). Barnaud et al. (2012) showed that HEV did not infect pigs when the temperature of experimentally contaminated food preparations had reached 71 °C for 20 min.

The survival of infectious HEV in fermented pork-containing sausages has rarely been investigated during non-thermal food processing, due to the challenges associated with HEV cultivation and detection from sausage matrices (Cook et al., 2017). In one study, Emmoth et al. (2017) spiked ready-made foods with a panel of viruses, including murine norovirus (MNV), to study HEV-like virus inactivation in common source pork-containing food matrices. MNV, which is commonly used to study human norovirus survival in food, was found to be rather tolerant to low pH after addition of lactic acid to liver and dry-cured ham.

Recently, Stunnenberg et al. (2023) studied inactivation of HEV in HEV-inoculated ready-made dry sausages. A semi-quantitative assay was used to detect infectious HEV-3c and HEV-3e in a cell culture medium and in extracts from inoculated pork products at various temperatures. HEV infectivity was still apparent in sausages after low-temperature treatments spanning a four-week period. In addition, Schilling-Loeffler et al. (2025) found only a slight decrease in infectious HEV titre in salami-like raw pork sausages over three weeks of curing. They also showed that the presence of HEV RNA in sausage, especially if thermally treated, cannot predict infectivity.

In the present study, MNV was inoculated into the meat batter from which salami-like sausages—dry, hard, ready-to-eat Finnish mettwurst—were prepared; the virus in sausage was then subjected to non-thermal sausage-making steps of the cold smoking process. The aim was to follow the survival of infectious viruses over four weeks of ripening. The survival of MNV and HEV in solution was also studied during the cold smoking process, and at RT without treatment. MNV was included in the study since it can be grown to relatively high titres and the cultivation technique was already established in the laboratory. The potential of viruses to survive in mettwurst was investigated using the experimental results obtained with MNV as data, which were modelled using Bayesian inference. The Bayesian methods provide the estimation of the uncertainty in the assessment.

## Materials and Methods

### Cells

RAW 254.7 gamma NO(-) cells (mouse monocyte-macrophage line, ATCC® CRL2278™) were used for MNV cultivation. The RAW cells were cultivated as previously described by Mosselhy et al. (2022). Dulbecco’s Modified Eagle Medium (DMEM) with high glucose and HEPES (Gibco™, 42,430,025) supplemented with 10% heat-inactivated Fetal Bovine Serum (FBS; Gibco™, 10,500,064) and 1% L-Glutamine–Penicillin–Streptomycin (GPS; Sigma-Aldrich, G1146) was used for these cells. 2.0 × 10^4^ RAW cells per well were used in 96-well cell plates (Thermo Scientific™, 161,093, Nunc™ MicroWell™ 96-Well), which were prepared one day before the samples were inoculated.

A549/D3 cells (adenocarcinoma human alveolar basal epithelial cells, received from Prof. Reimar Johne, Germany) and persistently infected A549 cells (used for virus stock preparation only, received from Prof. Reimar Johne, Germany) (Johne et al., 2014) were used for HEV cultivation and grown in flasks or 96-well plates as described earlier by Johne et al. (2016). Eagle’s Minimum Essential Medium (EMEM; Sigma-Aldrich®, M2279) supplemented with 1% nonessential amino acids (Gibco™, 11,140,050, 100X), 1% L-glutamine (Gibco™, 25,030,081, 200 mM), 0.5% gentamicin, and 10% FBS were used for A549/D3 cells. For persistently infected A549 cells, 5% FBS was used. All cells were cultivated in a cell culture incubator at 37 °C and with 5% CO_2_, except for persistently infected A549 cells, which were cultivated at 34.5 °C. Aliquoted batches of the cell lines were stored in liquid nitrogen.

### Viruses

MNV-1 (received from prof. Herbert W. Virgin, Washington University, USA) and HEV-3 strain 47832c (received from Prof. Reimar Johne, Germany) were used in this study. HEV stock was prepared using persistently infected A549 cells as previously described (Johne et al., 2016). The MNV stock was cultivated in the presence of DMEM with 1% GPS and 10% FBS, and was otherwise prepared as described by Park et al. (2014). Both virus stocks were stored in aliquots at − 72 °C. Undiluted viral stocks were used for virus inoculation (5% FBS in HEV stock, 10% FBS in MNV stock). The infectious titre of the HEV stock was 2.1 log_10_ TCID_50_/ml (tissue culture infectious dose) and that of the MNV stock was 7.6 log_10_ TCID_50_/ml. The RNA titre for HEV was 9.1 log_10_ genome copies (gc)/ml, and for MNV it was 9.8 log_10_ gc/ml.

### Experimental Overview

**Fig. 1:**
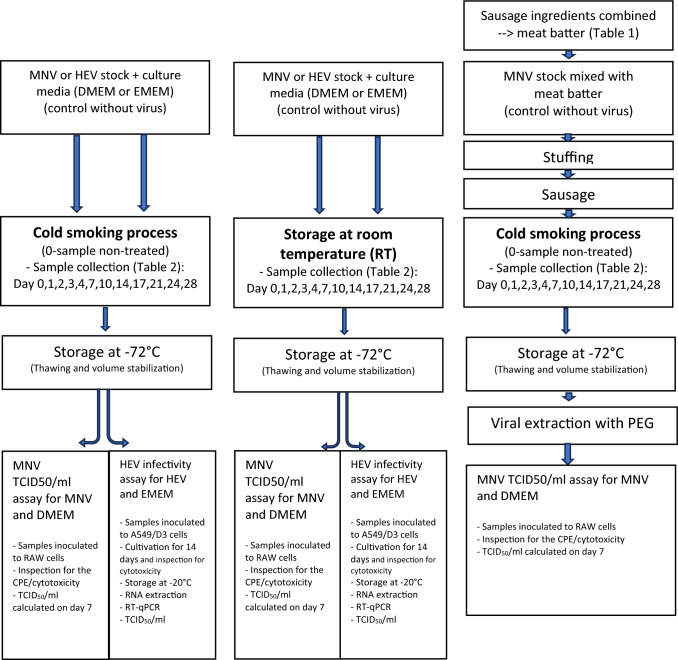
Methodology flowchart and the study setup

Inactivation of MNV was monitored in solution (cell culture medium) and in the sausage matrix while these sample types were subjected to a typical salami-like sausage manufacturing process in the ripening chamber, or alternatively, stored at RT for four weeks in solution. HEV inactivation was only monitored in solution during processing and RT storage. Overall methodology is presented in Fig. 1.

### Solution Samples and Preparation of Artificially Virus-Contaminated Salami-Like Sausages

For the solution samples, 1 ml of undiluted virus—either HEV or MNV—was aliquoted into 2 ml microcentrifuge tubes (PP, 72.691, Sarstedt). These tubes were used as (1) zero-time controls, or (2) taken as samples at different time points while the virus inactivation was monitored either (a) in solution during cold smoking, or (b) in solution at RT. Cytotoxicity was screened using media without any viruses.

The MNV-containing cold-smoked salami-like sausages (mettwurst) were prepared in an experimental production plant at the University of Helsinki. The recipe produced a typical mettwurst sausage with 66% meat content and 30% fat content (Table 1). 18 kg of meat batter per testing time was made with an automatic cutter (Seydelmann Stuttgart K 41), while the meat and lard were partially frozen. Approximately one half of the batch was used for control sausages without any virus, and the other half was inoculated with MNV. MNV stock (13.6 ml) was diluted with PBS (Phosphate Buffered Saline, 100 ml) and mixed into the meat batter (9.75 kg) used for viral sausages. The diluted virus was combined with the meat batter using a mixer (Metos Bear Mixer AR40, A/S Wodschow & Co.). Total dilution of MNV was approximately 1:725. A manual sausage stuffer (F. DICK 9 ltr.) was used to make the sausages, with a synthetic intestine (Viscofan REG 45 × 36 CLR, 421,000,015; calibre of 45–47 mm) as the casing. The sausages were approximately 10 cm in length and 4.5 cm in width.

**Table 1:** Recipe for the raw meat batter of mettwurst sausages

Ingredients	Percentage (%)	Mass per 18 kg (g)
Beef (boneless trimmed meat, fat < 10%)	33	5,940
Pork (boneless ham, fat < 5%)	33	5,940
Lard (fat 100%)	30	5,400
Starter culture^a^	0.02	3.6
Salt^b^	3	540
Glucose	0.3	54
Sodium nitrite (NaNO_2_ in 10% solution)	0.12	21.6
Ascorbic acid	0.06	10.8
Spice mix^c^	0.56	110.8

^a^Lyocarni BBL-97, freeze-dried culture, Sacco srl

^b^Percentage of salt in the final sausage product was approximately 4.1%

^c^Home-made, included mustard seed (48,7%, 54 g/110.8 g), paprika (17.9%, 19.8 g/110.8 g), white pepper (16.2%, 18 g/110.8 g), and black pepper (8.1%, 9 g/110.8 g)

### Cold Smoking Process

For the cold smoking process, a VEMAG Aditec MIC 2420 cold smoking chamber was used; the cold smoking program was similar to industrial processes with relevant temperature and relative humidity (RH) changes (Table 2). Cold-smoked solution (HEV or MNV samples, and cytotoxicity samples) and sausage samples were collected on day 0 (prior to starting the program), as well as days 1, 2, 3, 4, 7, 10, 14, 17, 21, 24, and 28 (Table 2). A smoking step (mild smoke made by burning alder wood chips, 30 min) was included in the program on days 1, 2, 3, and 4. On those days, the samples were collected after the smoking step.

**Table 2:** The cold smoking process and the sampling schedule

Week	Day	Step	Duration of the step (h)	Temperature of the chamber (°C) ^ab^	Relative humidity of the chamber (%)	Samples taken
1	0	Start				x
		Drying	2	23	85	
		Colour formation	10	24	90	
	1	Sweating	12	25	93	
		Cold smoking	0.5	24	93	x
		Ripening 1	11.5	24	92	
	2	Ripening 1	12	24	92	
		Cold smoking	0.5	23	92	x
		Ripening 2	11.5	22	90	
	3	Ripening 2	12	22	90	
		Cold smoking	0.5	20	88	x
		Ripening 3	11.5	20	87	
	4	Ripening 3	12	20	87	
		Cold smoking	0.5	20	87	x
	5	Ripening 4	24	18	82	
	6	Ripening 4	12	18	82	
		Ripening 5	12	16	75	
2–4	7–28	Ripening 5	516	16	75	x (days 7, 10, 14, 17, 21, 24, 28)

^a^At the start the temperature of the oven was on average 17.7 °C (SD 0.4 °C) and sausage 10.9 °C (SD 0.6 °C), and in the end 14.9 °C (SD 0.7 °C) and 15.8 °C (SD 0.0 °C), respectively)

^b^Maximal temperature for sausage was 25.8 °C (oven 27 °C). Minimum was 10.4 °C (oven 13 °C)

Two independent tests were conducted at different times, both of which included three parallel samples for MNV in solution and in sausage for the zero-time point and each experimental time point (Table 2). For HEV, one solution sample per time point was included in the first test, and two parallel samples in the second test. The caps of the solution samples were kept shut in a plastic tube holder (Fig. S1a) in the chamber, except during the smoking step, in which the tubes were opened. Therefore, only time, temperature, and smoking were affecting the samples during the cold smoking process. Virus-inoculated and control sausages were hung in the chamber in a similar manner, but from different sticks to avoid contamination (Fig. S1b). After each sampling, the solution samples were stored at − 72 °C immediately. The sausage samples were weighted, and two pieces (approximately 10 g each) were cut and stored at − 72 °C.

Temperature data from the chamber and three sausage samples were recorded with an external logger (Grant Data Logging, Grant Squirrel SQ800) that recorded temperature every 30 min.

### Experiments on the Stability of the Viruses at RT

The aliquoted MNV and HEV solutions were stored at RT for four weeks, and sampled to assess loss of virus infectivity over time. Two independent tests were performed at different times. In the first test, one sample was included per sample type (HEV, MNV, and cytotoxicity samples) per time point, while two parallel samples were included in the second test. The same sampling schedule (Table 2) was followed, and the samples were treated in the same way according to the matrix after collection. The average temperature of the laboratory was 21.8 °C (SD 0.5 °C) and RH 45.6% (SD 13.3%).

### Sample Preparation Prior to the Viral Infection

After the solution samples were thawed on ice, the volume was adjusted to 1.5 ml with the relevant media to counter the effect of potential volume loss due to drying. The sausage samples were also thawed on ice, and MNV was extracted with polyethylene glycol (PEG) precipitation, according to ISO 15216–1:2017 intended for soft fruit and leaf, stem, and bulb vegetables, with slight modifications. Approximately 6 g (± 0.05 g) of sausage was cut into 3–4 pieces and placed into a stomacher bag (Stomacher 400 Classic Strainer Bag) together with 15 ml PBS, and treated with the Easy MIX Blender (BioMérieux) for 60 s. Next, another 15 ml of PBS was added, and the bag with its contents was incubated in a shaker (150 oscillations min-1; IKA KS 260 Basic) for 20 min. The centrifugation steps (10,000 × g, 30 min, 4 °C) and addition of 5 × PEG/NaCl with an incubation for 1 h at 6 °C followed, according to the ISO method. Finally, 1 ml PBS was used to dissolve the precipitated virus in the pellet and the samples were centrifugated at 10,000 × g for 5 min at RT. All samples were stored on ice until viral cultivation.

### Viral Cultivation and Titration of HEV and MNV Samples

The TCID_50_ assay for the MNV samples was performed according to Mosselhy et al. (2022). Briefly, 100 µl of undiluted sample or the ten-fold dilution series prepared from this was added to RAW cells in 96-well plates to emptied wells. Each sample was tested in six parallel wells. After MNV inoculation, the cell culture plates were incubated at 37 °C and with 5% CO_2_ for 1 h. The inoculum was removed and replaced with 200 μl 10% DMEM per well. The plates were further incubated at 37 °C and with 5% CO_2_ for 1 week. Potential MNV infection was identified in the RAW 96-well cell plates by assessing the presence of viral CPE (cytopathic effect) with a light microscope.

Viral cultivation for HEV solution samples was performed as described by Johne et al. (2016) with some modifications. After sample inoculation, the 96-well A549 cell plates for HEV samples were incubated for 14 days at 37 °C and 5% CO_2_. The plates were then stored at − 20 °C until RNA extraction was performed. Potential HEV infection was identified from culture media supernatants collected from the plate wells. RT-qPCR was performed as described earlier by Loikkanen et al. (2020), with the difference that a 1:10 dilution of samples was used in the extraction, and the extracted RNA samples were not purified further. Briefly, 140 µl sample for RNA was extracted using a commercial kit (E.Z.N.A.® Viral RNA Kit, Omega Bio-tek, United States) according to the manufacturer’s instructions. Elution volume of 70 μl was used. The presence of HEV RNA was measured using QuantiTect Probe RT-PCR kit (Qiagen, Germany) for real-time one-step RT-qPCR, with a 5 µl sample of RNA in a 20 µl reaction. Primers and a probe were used according to Jothikumar et al. (2006). RT-qPCR was performed with a Rotor-Gene 3000 Instrument (Corbett Life Sciences, Sydney, Australia).

The limit for a sample to be considered as positive for HEV was at least 10^3^ gc/PCR reaction, which is equal to approximately 10^6^ gc/ml in culture media, as in our study on warm smoking (to be submitted, personal comment, E. Loikkanen).

### Calculation of the TCID_50_/ml and Statistical Analysis

The TCID_50_/ml and the limit of detection were calculated with M. Binder’s (Heidelberg University, Germany) TCID_50_ calculator, which uses the Spearman & Kärber algorithm (Hierholzer & Killington, 1996). For statistical analysis, IBM SPSS Statistics 29 programme (IBM) was used, and the differences were evaluated with one-way ANOVA and effect sizes by *η*2 (p < 0.05 was considered as statistically significant).

### Statistical Modelling

Bayesian statistical modelling was utilized to investigate the effect of the cold smoking process on the inactivation of MNV. The aim of the modelling was to represent the relationship between the process time and inactivation of the virus particles in sausage and in solution, as well as to describe the uncertainty in inactivation results. For the statistical model, experimental results obtained with MNV as data were used (six data values per time point). Data consisted of MNV concentration measurements (TCID_50_/ml) at the initial state, and eleven different time points from days 1 through 28. The model was performed for data on MNV loads in solution and in sausage. For comparison, virus inactivation was also modelled without treatment in solution at RT. One parametric log-linear model (Schaffner et al., 1997) and two parametric Weibull models (Peleg & Cole, 1998) were considered in describing inactivation during the process. The Weibull model was finally chosen because it provided a better fit for all datasets used in the study when deviance information criterion (DIC) values were compared. DIC is a metric used in Bayesian model selection, wherein the model with the smallest DIC value is preferred by the data (Spiegelhalter et al., 2002). Viral load reduction appeared to be faster during the first few days than later in the process, and the Weibull model is flexible to also handle nonlinear (concave and convex) forms to better describe this type of inactivation. The mathematical description of the Weibull model can be written as follows:

$$\log \left( {N_{t} } \right) = \log \left( {N_{0} } \right) - \left( {\frac{t}{\alpha }} \right)^{\beta },$$

where *t* is duration of the cold smoking treatment in days, *N*_0_ is the initial number of viruses, and *N*_t_ is the expected number of viruses at time *t*. The parameter alpha denotes scale and beta shape parameter of the Weibull model. The time in days required to achieve 1 log_10_ reduction (D value) in viral load equals the value of the scale parameter in this model form. Conventional uninformative (uniform) prior distributions were assigned for the scale and shape parameters in the Bayesian model. The model for the observed decrease (*Y*_*i,t*_) was defined as Log(*Y*_*i,t*_) ~ Normal(log(*N*_*t*_), τ), where *i* denotes experiment and τ is the precision parameter, which is inverse of the variance parameter (*τ* = 1/*σ*^2^). Conventional uninformative prior distribution was placed for the precision parameter by giving uniform distribution for standard deviation parameter (*σ*). The computation of the model was performed using OpenBUGS software (Lunn et al., 2000, 2013). The results were based on 50,000 iterations after the burn-in phase of 10,000 iterations, in which the convergence of the MCMC chain was reached.

## Results

### Viral Inactivation in Solution During the Cold Smoking Process and at RT

Infective MNV was detected throughout the duration of the cold smoking process until day 28, when the virus was in solution (Table 3). During the first and second week, the infectivity decreased the most, by 2.2 log_10_ TCID_50_/ml and 1.9 log_10_ TCID_50_/ml (total 4.1 log_10_ TCID_50_/ml reduction after two weeks of cold smoking process), respectively. During the third week, infectivity decreased by only 0.2 log_10_ TCID_50_/ml (total 4.3 log_10_ TCID_50_/ml reduction). At RT, infective MNV was also detected in solution throughout the entire test period. The MNV infectivity decreased significantly more slowly at RT than during the cold smoking process (*p* < 0.05; log_10_ reduction of 1.8 vs. 4.7 TCID_50_/ml after four weeks at RT or during the cold smoking process, respectively).

**Table 3:** MNV titre and inactivation results in sausage and in solution during the cold smoking process and storage at room temperature. Results are shown as mean values in log_10_ TCID_50_/ml. Standard deviations are visible in brackets

Day	MNV in solution				MNV in sausage	
	Cold smoking process		Room temperature storage		Cold smoking process	
	Titre	Inactivation^a^	Titre	Inactivation^a^	Titre	Inactivation^a^
0	7.6 (0.4)	–	7.7 (0.2)	–	4.6 (0.3)	–
1	7.1 (0.4)	0.4 (0.6)	7.7 (–)	0.2 (–)	4.0 (0.6)	0.7 (0.8)
2	6.7 (0.7)	0.8 (0.6)	7.7 (–)	0.2 (–)	3.8 (0.2)	0.9 (0.4)
3	6.2 (0.8)	1.3 (0.7)	7.8 (–)	0.0 (–)	3.6 (0.2)	1.1 (0.3)
4	6.2 (0.5)	1.3 (0.4)	7.4 (0.1)	0.4 (0.1)	3.4 (0.25)	1.3 (0.3)
7	5.4 (0.9)	2.2 (0.6)	6.8 (0.2)	0.9 (0.3)	3.6 (0.5)	1.1 (0.6)
10	4.8 (1.6)	2.8 (1.6)	6.7 (0.1)	1.0 (0.2)	3.2 (0.1)	1.4 (0.2)
14	3.5 (1.6)	4.1 (1.5)	6.7 (0.3)	1.0 (0.4)	2.9 (0.7)	1.6 (0.5)
17	3.6 (1.8)	4.0 (1.7)	6.5 (0.3)	1.1 (0.1)	2.9 (0.2)	1.6 (0.3)
21	3.4 (1.8)	4.3 (1.8)	6.2 (0.4)	1.5 (0.4)	3.0 (0.3)	1.5 (0.2)
24	3.0 (1.4)	4.6 (1.4)	5.9 (0.3)	1.7 (0.3)	2.8 (0.2)	1.7 (0.4)
28	3.0 (0.9)	4.7 (0.9)	5.8 (0.2)	1.8 (0.2)	2.7 (0.5)	1.9 (0.5)

^a^Inactivation means here the virus titre difference between the titre at zero-point (day 0) and the titre on the specific day, and it has been calculated from the actual values rather than mean values

The individual virus titre values of each sample and the zero-point, both cultivated on the same day, were compared (rather than the mean values shown in the table versus the zero-point)

HEV infectivity dropped below the detection threshold on the third day (maximal 1.1 log_10_ TCID_50_/ml reduction) during the cold smoking process (Table 4). At RT, HEV dropped below detection threshold on the seventh day. HEV survived for a shorter period than MNV, which was likely a result of the clear difference in the titres between the two viruses. Due to the low titre of HEV, only MNV could be monitored in sausages.

**Table 4:** HEV titre and inactivation results in solution during the cold smoking process and storage at room temperature. Results are shown as mean values in log_10_ TCID_50_/ml. Standard deviations are visible in brackets

Day	Cold smoking process		Room temperature storage	
	Titre	Inactivation^a^	Titre	Inactivation^a^
0	1.7 (0.2)	–	1.7 (0.4)	–
1	1.3 (0.3)	0.4 (0.4)	1.4 (0.6)	0.3 (0.2)
2	0.9 (0.3)	0.8 (0.4)	1.3 (0.4)	0.4 (0.1)
3	< 0.7 (0.0)	> 1.1 (0.2)	1.0 (0.3)	0.7 (0.1)
4	–	–	0.8 (0.3)	0.9 (0.2)
7	–	–	< 0.7 (0.0)	> 1.1 (0.4)

^a^Inactivation means here the virus titre difference between the titre at zero-point (day 0) and the titre on the specific day, and it has been calculated from the actual values rather than mean values

The individual virus titre values of each sample and the zero-point, both cultivated on the same day, were compared (rather than the mean values shown in the table versus the zero-point)

### MNV Inactivation in Artificially Virus-Contaminated Sausages during the Cold Smoking Process

MNV survived in artificially contaminated sausages throughout the cold smoking treatment (Table 3). The MNV infectivity decreased the most during the first week of treatment (reduction of 1.1 log_10_ TCID_50_ml). During the last three weeks, the decrease in MNV infectivity was limited (maximal reduction of 0.5 log_10_ TCID_50_/ml per week, total 1.9 log_10_ TCID_50_/ml after four weeks of cold smoking process).

### Parameters of the Cold Smoking Process

Temperatures during the cold smoking process were similar between test times. The temperature of the sausages closely followed the chamber’s set temperature (Table 2). The weight of the sausages varied between 150 and 250 g at the start of the process, and decreased with longer exposure to the cold smoking program. At the end of the process, the sausages weighed 100–130 g, corresponding to approximately 26% of weight loss.

### Modelling of MNV Inactivation

**Fig. 2:**
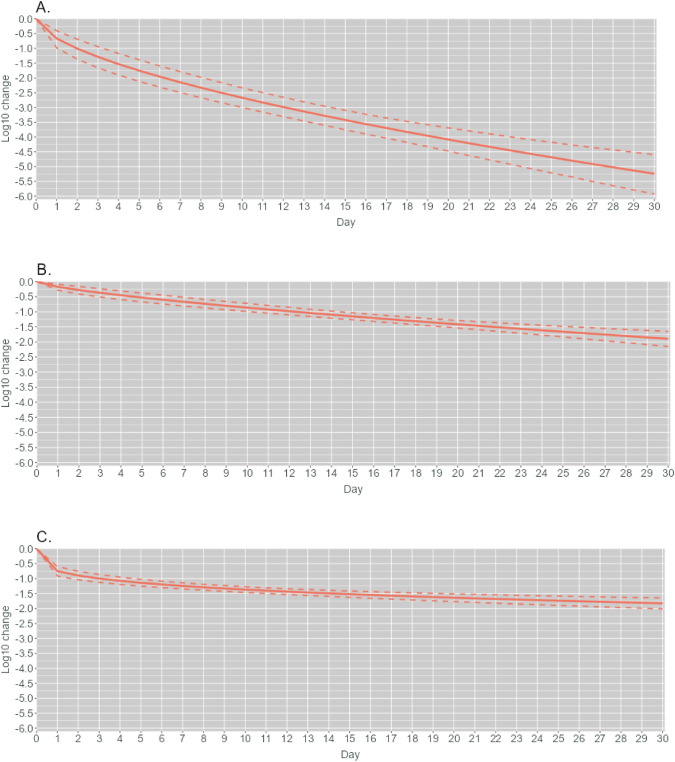
Mean and 95% CI for the expected inactivation of murine norovirus **A** in solution during the cold smoking treatment, **B** in solution at room temperature, **C** in salami-like sausage after the cold smoking treatment. The estimated inactivation is shown relative to the duration of the treatment

The MNV inactivation curves based on modelling are shown in Fig. 2 (see also Table S1 for detailed values). The estimate refers to posterior mean, and 95% CI refers to the credible interval (Bayesian confidence interval) that describes the uncertainty related to the result. The MNV load reduction was estimated to be remarkably faster during the first few days of the cold smoking treatment compared to the later days. A similar concave shape was observed in the modelled inactivation curves of the treated viruses in solution, and especially in sausage (Fig. 2a and c). After 30 days, around 5.2 log_10_ (95% CI 4.6–5.9) expected reduction of MNV was achieved in solution during the cold smoking treatment; at RT, around 1.9 log_10_ (95% CI 1.7–2.1) expected reduction was reached in solution (Fig. 2b). Around 1.8 log_10_ (95% CI 1.6–2.0) expected MNV reduction was achieved in sausage. Thus, the values vary only slightly from the experimental inactivation values reported in Table 3.

The D value for MNV, i.e. the time required to inactivate 90% of the viruses, was estimated to be 2.2 days (95% CI 1.1–3.4) in solution when the cold smoking treatment was used. Without cold smoking, virus inactivation was estimated to be much slower, with a D value of around 12 days (95 CI 10.1–14.5). For MNV in sausage, the D value estimate was 3.1 days (95% CI 1.6–4.8) when the cold smoking treatment was used.

In general, it was observed that the uncertainty increased towards the end of the monitoring in all three setups. The uncertainty was most prominent when MNV in solution underwent the cold smoking treatment, even from the beginning. In this scenario, 99.999% MNV might have been inactivated by the end of the experiment, if uncertainty was considered (lower bound). However, there was no indication that MNV in sausage would have been inactivated in the near future, even when the uncertainty was considered.

## Discussion

Viruses that contaminate food can be inactivated at high temperatures, but pork-containing salami-like sausages are processed non-thermally, and may thus spread zoonotic HEV. In the present study, experimenting with infectious HEV was found to be challenging. For this reason, HEV inactivation could not be monitored in the artificially virus-contaminated sausages. However, monitoring MNV inactivation was successful. The main observation was that although some MNV inactivation occurred over the four-week period, infectious MNV was still detected in sausages—mettwurst—after they had been subjected to salami-like sausage manufacturing treatment involving cold smoking.

Thus far, research has mainly focussed on thermal inactivation of HEV based on literature, as reviewed by Cook and van der Poel (2015), EFSA (2017), and Johne et al. (2024). Treatments over 50 °C have usually been performed for viruses in solution (cell culture supernatant) (Imagawa et al., 2018; Johne et al., 2016), as well as in food matrices, such as wild boar liver suspension (Schielke et al., 2011), liver homogenate (Feagins et al., 2008), paté (Barnaud et al., 2012), and minced pork meat (Imagawa et al., 2018), or in stool preparation (e.g. Emerson et al., 2005; Huang et al., 1999). In addition, the high persistence of HEV at 4 °C and when stored frozen has been studied (Monini et al., 2024).

Notably, there is practically no recent data on the tolerance of HEV or other viruses for cold smoking in sausages (EFSA, 2017). Most of the original studies—even the early ones for ASFV—describe virus inactivation in other types of salami-like sausages, but not in mettwurst, the processing of which involves smoking treatment. Smoking is mentioned in some publications, but usually only in the introduction or discussion (e.g. Edwards, 2000; Hinrichs et al., 2024; Petrini et al., 2019) or reviewed by EFSA (2017).

The sausage-making process used in the present study consisted of a multi-step protocol, where daily cold smoking intervals at a maximum temperature of 25 °C were used during the first week, whereas a constant temperature of 16 °C and RH 75% prevailed throughout the following three weeks. When MNV in solution (cell culture medium) was treated with the same treatment process as sausages, the virus titre decreased by several log_10_ units, however, infectious virus was still detectable after the 28-day treatment. Furthermore, the MNV in solution was found to inactivate faster during the cold smoking process than at RT storage. The clear difference in virus inactivation rates with and without processing suggests that the cold smoking intervals have some inactivation efficiency for viruses when the virus is treated in solution. While samples stored at RT were protected in closed tubes at all times, they were kept open during the smoking exposure. To date, no other studies have been published in which viruses in solution were exposed to cold smoking. Regarding other microbes, mild inhibition of smoke for growth is due to antimicrobial compounds such as phenols, aldehydes, acids, or reduction of water activity, mild surface drying, or alteration of pH (Ogbadu, 2014). However, none of these might affect viruses. More experimental studies are required to generate data on the effect of these parameters.

There are not many studies on MNV inactivation over a long-term storage of four weeks at RT (15–25 °C), and seemingly, none in which a similar salami-like sausage manufacturing was applied. Hence, the results of this study can only be compared with the experiments in which MNV was stored in solution at a constant RT. Wu et al. (2023) detected a maximum decrease of 1.5 log_10_ (PFU/ml, Plaque Forming Unit) over 28 days at 19 and 25 °C. Similarly, in the study of Zhao et al. (2021), MNV titre decreased by less than 2 log_10_ PFU in bottled drinking water over 40 days at 20 °C; these results are in line with the MNV results in DMEM solution of the present study at RT (1.8 log_10_ reduction, TCID_50_). Seo et al. (2012) monitored MNV inactivation in DMEM and found that the decrease was 2 log_10_-units PFU/ml in 12 days at RT (24 °C). Compared to the study of Seo et al. (2012), the virus reduction rate of the present study was slower in solution at RT. Overall, a common result in the above-mentioned studies was that infectious MNV could still be detected after four weeks when a high virus load in solution was used in the inactivation experiments.

Johne et al. (2016) have shown that titre of HEV in solution decreased over 3 log_10_ ffu/ml, when stored at RT (21 °C) for 28 days. Compared to the present study, HEV in the study of Johne et al. (2016) was less resistant than MNV in solution, although remnants of infectious virus were detected until day 28 in both studies. In addition, the difference in the original virus titres might partly explain the results. In the present study, the very low-titre HEV stock preparation became inactivated even faster than that at RT.

Stunnenberg et al. (2023) monitored HEV inactivation in cell culture medium and in HEV-inoculated ready-made dry sausage, without any cold smoking process, at various temperatures, including RT (21 °C), over time. They showed (in their Figs. 1 and 2) that the titre of HEV-3c/e decreased by more than 3 log_10_ ROPE (region of practical equivalence) in solution, whereas the decrease was less than 2.5 log_10_ ROPE in sausage over four weeks at RT. In line with that, in the present study, MNV titre also decreased in solution, by 1.8 log_10_ TCID_50_/ml at RT; MNV inactivation was not monitored in sausage at RT. However, MNV inactivation was monitored in solution and sausage during the cold smoking treatment, during which the inactivation rate was also found to be slower in sausage than in solution. This observation is in accordance with the Stunnenberg et al. (2023) study, despite the obvious differences between the two studies.

One reason for the modest MNV and HEV reduction in the sausage matrices of the two studies as compared to those in solution might be the presence of complex ingredients, as well as high protein and fat loads in the sausage matrix/meat batter. Similar observations on the influence of matrices have been reported earlier in the thermal treatment studies, in which increased virus resistance to heat treatment was observed when viruses were embedded within tissues or other matrices (e.g. liver or faeces) as compared to when they were in solution (EFSA, 2017). A minimal decrease of 0.45 log_10_ in 3 days at 22 °C was found in the study of Schielke et al. (2011) when HEV survival was investigated in homogenates of infected wild boar liver. However, pretreatment RT-PCR was used in that study, instead of virus cultivation in cells.

During the manufacturing of sausages, there are multiple variables to consider, e.g. cold smoking and fermenting/ripening treatment, the presence of possible inactivating ingredients such as nitrate, and changes in RH and pH, and in the sausage consistency. In the study of Straube et al. (2011), ECHO virus was highly stable in the presence of sodium nitrite and D/L-lactic acid (sausage ingredients) at RT, whereas feline calicivirus was resistant at 4 °C. HEV was also reported to remain stable at high salt concentrations (Wolff et al., 2020a), and at various pH conditions (2–9) (Wolff et al., 2020b). Emmoth et al. (2017) also found MNV in ham to be rather resistant to low pH (pH 3.1–3.7, 10 min; 0.55–2.2 M, 4–16% lactic acid), as did Seo et al. (2012) when MNV was in solution (pH 2, pH 4). Wolff et al. (2020b) observed that HEV infectivity decreased in the presence of 2% sodium chloride with 0.015% sodium nitrite in PBS, with a significant reduction (less than one log_10_) after two and four days of incubation at 22 °C as compared to PBS only. According to Seo et al. (2012), MNV better tolerated low salt concentrations compared to high ones (0.3% vs. 3.3–6.3%). Comparison between these results and those of the present study is difficult, since inactivation of viruses in all of the above-mentioned studies has been examined with the virus in solution matrix, or because the virus was added to ready-made ham (Emmoth et al., 2017) rather than to the meat batter that went through the salami-like sausage manufacturing, as in the present study. In line with those studies, lactic acid and salt concentrations—which were low in our sausages in the beginning but increased during the process—likely did not influence the MNV inactivation rate. However, further detailed experiments are required before drawing conclusions. It was clear that the virus species used in the studies have an influence as well.

In this study, the virus inactivation curves of MNV in solution at RT and in sausage during treatment showed dissimilarities in their shapes during the first week, despite the similar total MNV decrease of 1.8 and 1.9 log_10_ TCID_50_ after four weeks. Specifically, MNV load in sausage decreased until day four at a rate that was almost four times faster than that in solution at RT, whereas the rate remained relatively constant at RT for four weeks. The fastest virus load decrease in the sausage matrix coincided with the smoking phase in the cold smoking treatment. This phenomenon may indicate that some of the inactivating effect occurred during the cold smoking treatment, but the protective presence of fat or other sausage ingredients (or lower temperatures) partly compensated for the overall inactivation rate. The bi-phasic virus inactivation curves are frequently obtained after thermal treatments (Doyle, 2010).

This study has some limitations worth noting. Although the experiments using MNV were successful, it was not possible to test HEV inactivation in sausage matrix due to the low-titre virus stock preparation. This issue also impacted the experiments performed with HEV in solution. There was remarkable variability in inactivation rates between different experiments, and the number of experimental data values that could be used in the modelling was relatively low. This uncertainty related to the expected inactivation rates was provided by the modelled 95% credible intervals. In the future, more repetitions and independent trials are required to collect more evidence.

The experiments of this study showed that manufacturing salami-like cold-smoked sausages had a rather modest inactivating effect on MNV in sausages. Based on the persistence of MNV in the sausage matrix, it is possible that also HEV, if present in high loads in sausage, would not be inactivated by the process in a worst-case scenario. This would be in line with the results of Stunnenberg et al. (2023) and Schilling-Loeffler et al. (2025), who showed that HEV-inoculated sausage remained infectious even when food-processing steps for dried sausages were mimicked and during curing of salami-like pork sausages. There is a paramount need to continue these kinds of experiments to collect more data to understand the persistence of HEV in pork products.

Early studies performed with several porcine viruses (ASFV, classical swine fever, PRRSV etc.) have shown that the resistance of different viruses varies in salami-like sausages, as well as in different pork products, as reviewed by Farez and Morley (1997). The differences in results obtained in the present study when MNV inactivation was monitored in solution or in sausage matrix during cold smoking clearly indicate that it is important to experiment using sausage matrix rather than deduce results based on studies made using viruses in solution. This pilot study underlines the importance of establishing convenient, less time-consuming methods for infectious HEV detection in food. When reliable tests have been established, it is possible to study several recipes and manufacturing processes, and thus determine their influence in virus survival. However, before sufficient methods are available, testing any virus—even MNV—may provide relevant information.

## Conclusions

This study demonstrated that the salami-like sausage (mettwurst) manufacturing used was not efficient enough to inactivate MNV in sausage, which is a challenging food matrix. The virus inactivation could be monitored in artificially MNV-contaminated salami-like pork sausages during the manufacturing. The non-thermal cold smoking process that was used in this study did not inactivate all MNV from the sausages. However, it seems that both the MNV and low-titre HEV loads in solution decrease faster during the cold smoking process than during storage at RT. Further studies are required to investigate the survival of high-titre HEV in pork-containing salami-like sausages.

## Supplementary Information

Below is the link to the electronic supplementary material.

Supplementary file1 (DOCX 678 KB)

## Data Availability

No datasets were generated or analysed during the current study.
