# Supplementary material for: Effectiveness of Cold Smoking on Inactivating Murine Norovirus in Salami-Like Pork Sausages (Mettwurst), and Hepatitis E Virus and Murine Norovirus in Solution
Source: Food Environ Virol. 2025 Jan 10;17(1):17. doi: 10.1007/s12560-024-09631-1 (PMC11723849; doi:10.1007/s12560-024-09631-1)

**Supplementary Material**

**Effectiveness of Cold Smoking on Inactivating Murine Norovirus in Salami-Like Pork Sausages (Mettwurst), and Hepatitis E Virus and Murine Norovirus in Solution**

Emil Loikkanen^1^, Antti Mikkelä^2^, Suvi Joutsen^2^, Pirkko Tuominen^2^, Leena Maunula^1^

*^1^Department of Food Hygiene and Environmental Health, Faculty of Veterinary Medicine, University of Helsinki, Helsinki, Finland*

*^2^Risk Assessment Unit, Laboratory and Research Department, Finnish Food Authority, Helsinki, Finland*

**Supplementary Information**

**Table S1** Mean and 95% CI for the expected inactivation of murine norovirus in different conditions and time points.

| Duration (days) | Sausage (CST ^1^) | Solution (CST) | Solution (RT^2^) |
| --- | --- | --- | --- |
| 1 | -0.75 (-0.59 to -0.91) | -0.66 (-0.40 to -0.96) | -0.17 (-0.08 to -0.29) |
| 2 | -0.89 (-0.75 to -1.04) | -1.00 (-0.69 to -1.34) | -0.28 (-0.16 to -0.42) |
| 3 | -0.99 (-0.86 to -1.12) | -1.28 (-0.94 to -1.63) | -0.37 (-0.24 to -0.52) |
| 4 | -1.07 (-0.95 to -1.19) | -1.52 (-1.17 to -1.88) | -0.45 (-0.31 to -0.61) |
| 5 | -1.14 (-1.02 to -1.25) | -1.74 (-1.39 to -2.10) | -0.53 (-0.38 to -0.68) |
| 6 | -1.19 (-1.08 to -1.30) | -1.95 (-1.59 to -2.29) | -0.60 (-0.45 to -0.75) |
| 7 | -1.24 (-1.14 to -1.34) | -2.14 (-1.79 to -2.48) | -0.67 (-0.52 to -0.82) |
| 8 | -1.29 (-1.18 to -1.39) | -2.32 (-1.98 to -2.65) | -0.74 (-0.59 to -0.88) |
| 9 | -1.33 (-1.23 to -1.43) | -2.50 (-2.16 to -2.82) | -0.80 (-0.65 to -0.94) |
| 10 | -1.36 (-1.26 to -1.46) | -2.66 (-2.33 to -2.98) | -0.86 (-0.72 to -1.00) |
| 11 | -1.40 (-1.30 to -1.50) | -2.82 (-2.50 to -3.14) | -0.92 (-0.78 to -1.05) |
| 12 | -1.43 (-1.33 to -1.53) | -2.98 (-2.66 to -3.29) | -0.98 (-0.85 to -1.11) |
| 13 | -1.46 (-1.36 to -1.56) | -3.13 (-2.81 to -3.44) | -1.04 (-0.91 to -1.16) |
| 14 | -1.49 (-1.38 to -1.59) | -3.28 (-2.95 to -3.59) | -1.10 (-0.97 to -1.22) |
| 15 | -1.52 (-1.41 to -1.62) | -3.42 (-3.09 to -3.74) | -1.15 (-1.03 to -1.27) |
| 16 | -1.54 (-1.43 to -1.66) | -3.56 (-3.22 to -3.89) | -1.21 (-1.08 to -1.32) |
| 17 | -1.57 (-1.45 to -1.68) | -3.69 (-3.34 to -4.04) | -1.26 (-1.14 to -1.38) |
| 18 | -1.59 (-1.47 to -1.71) | -3.83 (-3.46 to -4.18) | -1.31 (-1.19 to -1.43) |
| 19 | -1.62 (-1.49 to -1.74) | -3.95 (-3.58 to -4.33) | -1.36 (-1.24 to -1.49) |
| 20 | -1.64 (-1.51 to -1.77) | -4.08 (-3.68 to -4.48) | -1.41 (-1.28 to -1.54) |
| 21 | -1.66 (-1.52 to -1.79) | -4.21 (-3.79 to -4.62) | -1.46 (-1.33 to -1.60) |
| 22 | -1.68 (-1.54 to -1.82) | -4.33 (-3.89 to -4.77) | -1.51 (-1.37 to -1.66) |
| 23 | -1.70 (-1.56 to -1.84) | -4.45 (-3.98 to -4.91) | -1.56 (-1.41 to -1.71) |
| 24 | -1.72 (-1.57 to -1.87) | -4.57 (-4.08 to -5.05) | -1.61 (-1.45 to -1.77) |
| 25 | -1.74 (-1.58 to -1.89) | -4.69 (-4.17 to -5.20) | -1.66 (-1.48 to -1.83) |
| 26 | -1.76 (-1.60 to -1.92) | -4.80 (-4.26 to -5.34) | -1.71 (-1.52 to -1.89) |
| 27 | -1.77 (-1.61 to -1.94) | -4.92 (-4.35 to -5.49) | -1.75 (-1.55 to -1.95) |
| 28 | -1.79 (-1.62 to -1.96) | -5.03 (-4.44 to -5.63) | -1.80 (-1.59 to -2.01) |
| 29 | -1.81 (-1.63 to -1.99) | -5.14 (-4.52 to -5.78) | -1.85 (-1.62 to -2.08) |
| 30 | -1.82 (-1.65 to -2.01) | -5.25 (-4.61 to -5.91) | -1.89 (-1.65 to -2.14) |

^1^ cold smoking treatment

^2^ room temperature

**Figure legends**

**Fig. S1** The setup of A) solution samples and B) salami-like sausages (mettwurst) in the cold smoking chamber at the start of the cold smoking process. On the left side are the virus-contaminated sausages, and on the right side are the control sausages without added virus.

**Fig. S1**


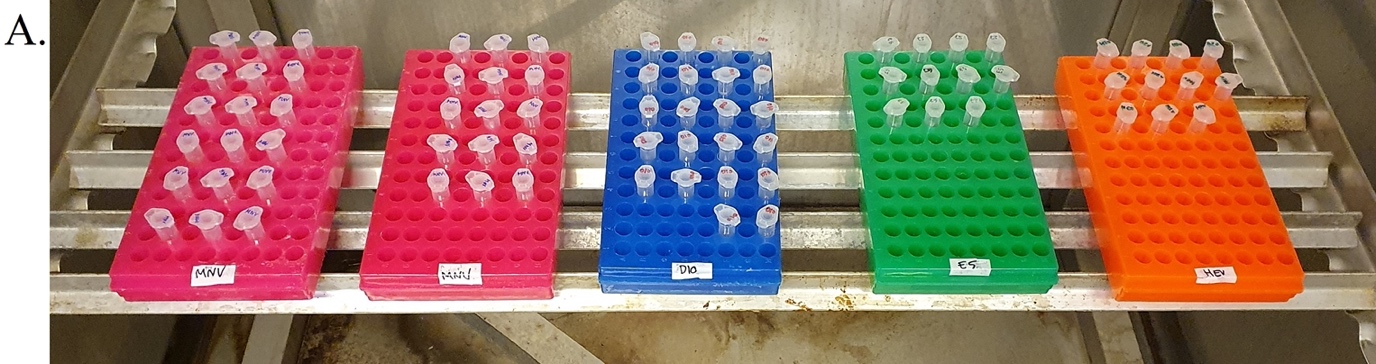

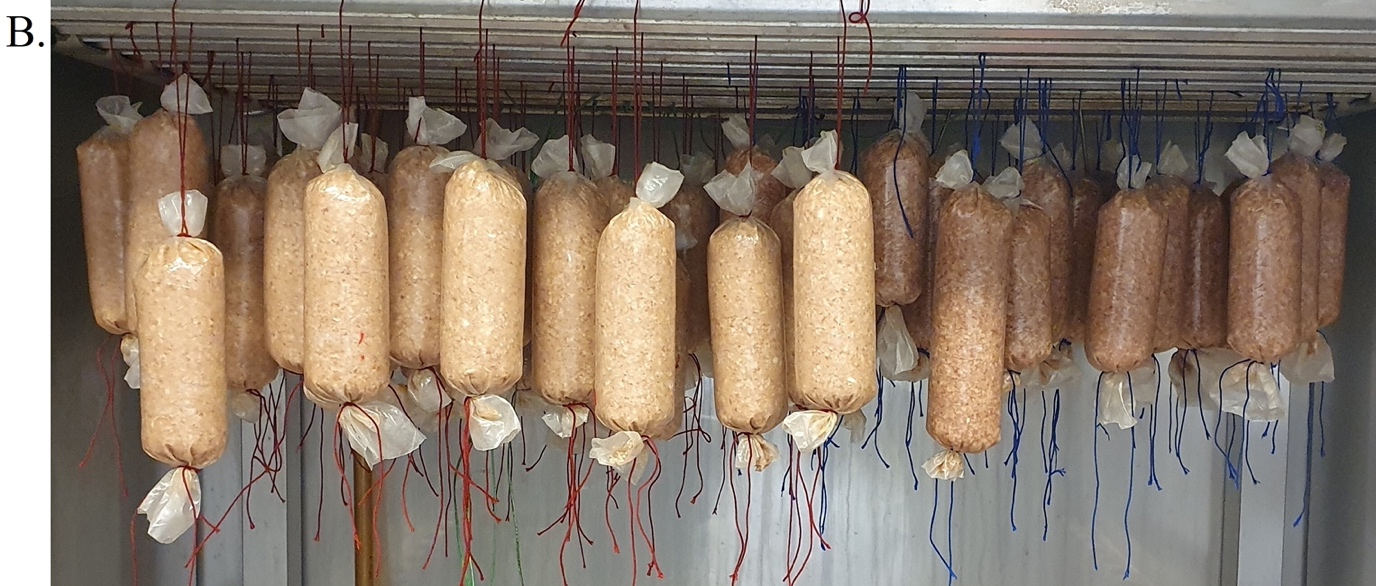

Supplement: Supplementary file 1 — Supplementary file1 (DOCX 678 KB) [file 12560_2024_9631_MOESM1_ESM.docx]
